# The East Palestine Disaster: The Potential Toxic Effects of Vinyl Chloride Exposure on Cardiovascular Health

**DOI:** 10.7759/cureus.46835

**Published:** 2023-10-11

**Authors:** Hanad Bashir, Gauranga Mahalwar, Timothy Henry

**Affiliations:** 1 Cardiovascular Medicine, The Christ Hospital, Cincinnati, USA; 2 Internal Medicine, Cleveland Clinic Akron General, Akron, USA

**Keywords:** public health, population surveillance, industrial accidents, atherogenic effects, insulin resistance, cardiovascular disease risk, vinyl chloride

## Abstract

This editorial explores the potential link between vinyl chloride (VC) and cardiovascular diseases, specifically in the context of a recent train derailment in East Palestine, Ohio. The primary finding of this article suggests a likely increased risk of cardiovascular factors associated with vinyl chloride exposure. This underscores the importance of proactive risk management and enhanced population monitoring. Together, these findings highlight the need for timely interventions to address cardiovascular health concerns in exposed populations.

## Editorial

Recently, a train derailment in East Palestine, Ohio, brought the impact of industrial accidents on healthcare into focus. The release of toxic chemicals, such as vinyl chloride (VC), has been linked to a variety of adverse health outcomes. Almost 1.1 million pounds of vinyl chloride, a toxic and flammable gas associated with respiratory issues, liver injury, and cancer, were released into the environment from five tankers [1]. Vinyl chloride is used in the production of polyvinyl chloride and was also utilized as an aerosol propellant and refrigerant prior to the 1970s. This gas has the potential to generate dioxins and derivatives, notorious carcinogens that can persist in the air, soil, and water for many years. Exposure to these chemicals, both acutely and chronically, can lead to serious negative health outcomes, including an increased risk of cardiovascular disease. Local medical facilities were reportedly under-equipped, and the vulnerable population was not well informed about the health consequences of the toxic leakage [1].

Kudaeva and Rukavishnikov reported that workers exposed long-term to toxic chemicals, including vinyl chloride, may experience atherogenic effects by altering cholesterol metabolism. Specifically, vinyl chloride exposure initially lowers high-density lipoproteins (HDL), increasing atherogenicity, followed by elevated low-density lipoprotein (LDL) levels and cholesterol levels. Chronic exposure to vinyl chloride and its toxic combustion products also stimulates very low-density lipoproteins' involvement in the atherogenic process. These pro-atherogenic effects have the potential to accelerate early atherosclerosis, arterial hypertension, and ischemic heart disease [2].

Furthermore, a study by Zelko et al. provides valuable insight into the relationship between exposure to vinyl chloride and the development of cardiovascular disease in mice. The study found that high exposure to vinyl chloride was associated with a high prevalence of steatohepatitis, insulin resistance, and proinflammatory cytokines [3]. The well-established link between insulin resistance and cardiovascular disease risk was evident. Hepatic insulin resistance can elevate several factors associated with high cardiovascular disease risks, such as blood glucose levels, c-reactive protein, LDL, plasminogen activator inhibitor 1, fibrinogen, and low HDL. Impaired glucose tolerance is also a prominent risk factor for heart disease. Thus, vinyl chloride-induced insulin resistance could potentially induce or exacerbate cardiovascular disease risk in humans [3].

In a comprehensive cohort analysis involving 1658 male workers in Italy, it was observed that exposure to VC was associated with a substantially elevated risk of cardiovascular disease (RR = 2.25) [4]. These findings underscore the critical importance of understanding the complex interplay between genetic factors and vinyl chloride exposure when assessing the health risks associated with this hazardous compound [4].

Given the limited available data, it appears reasonable for populations exposed to vinyl chloride to undergo aggressive risk factor modification and increased population surveillance for cardiovascular events. Aggressive risk factor modification can involve implementing measures aimed at reducing or eliminating known risk factors associated with cardiovascular disease in individuals exposed to vinyl chloride. This may include lifestyle modifications such as smoking cessation, dietary improvements, regular physical activity, and the management of conditions that increase cardiovascular risk. Other ways include increasing population surveillance; examples can include enhancing regular health check-ups, screening, and data collection to detect early signs of cardiovascular disease. It is important to efficiently mitigate the health hazards stemming from exposure, ranging from the protection of industrial workers to proactive risk factor modification and surveillance of populations exposed to this chemical during industrial accidents.

Further research is also needed to better understand the mechanisms by which vinyl chloride exposure leads to cardiovascular disease and to develop effective strategies for countering its harmful effects. The federal government has taken several steps to ensure safety and address the concerns of the residents of East Palestine. The Environmental Protection Agency is monitoring air quality and sampling water and soil to ensure the protection of the residents. The Department of Health and Human Services and the Centers for Disease Control and Prevention are conducting public health testing and assessments in the area to ascertain any long-term effects of vinyl chloride exposure. The government has also issued informational documents to increase awareness among the residents regarding the effects of vinyl chloride [5]. With the limited data on the effects of vinyl chloride on the cardiovascular system, close monitoring of the exposed population is required to ensure the limitation of adverse health events.
